# Possible Association of Hysterectomy Accompanied with Opportunistic Salpingectomy with Early Menopause: A Retrospective Cohort Study

**DOI:** 10.3390/ijerph191911871

**Published:** 2022-09-20

**Authors:** Pei-Chen Chen, Pei-Chen Li, Dah-Ching Ding

**Affiliations:** 1 Department of Obstetrics and Gynecology, Hualien Tzu Chi Hospital, Buddhist Tzu Chi Medical Foundation, Tzu Chi University, Hualien 97004, Taiwan; 2 Institute of Medical Sciences, Tzu Chi University, Hualien 97004, Taiwan

**Keywords:** opportunistic salpingectomy, ovarian function, menopause, hysterectomy, cancer

## Abstract

Opportunistic salpingectomies (OSs) are concurrently performed with hysterectomies to prevent epithelial ovarian cancer. This study aimed to investigate the correlation between OS and early menopause in females who have undergone hysterectomies. This was a retrospective cohort study involving 79 females who had undergone a hysterectomy, with or without an OS, between January 2007 and December 2015. Their ages at surgery, at menopause, and the lengths of time from surgery to menopause were compared. An OS had been performed in 54 and not performed in 25 of the enrolled patients, comprising the OS and non-OS groups. Body mass index was significantly higher in the OS group (OS: 25.27 ± 4.17 vs. non-OS: 22.97 ± 3.27, *p* = 0.01). Additionally, menopausal sleep problems were more prevalent in the OS group than in the non-OS group (41% vs. 12%, *p* = 0.01). Notably, the time from surgery to menopause was significantly shorter in the OS group than in the non-OS group (OS: 1.84 ± 1.85 vs. non-OS: 2.93 ± 2.43, *p* = 0.031). After adjusting the covariates, the OS group was associated with a significantly shorter period between surgery and menopause (*p* = 0.029). In conclusion, these results showed that a hysterectomy plus an OS might cause earlier menopause than a hysterectomy only. An OS should be preoperatively discussed with patients regarding the possibility of early menopause. The findings of this study require further large-scale investigations to reinforce the results.

## 1. Introduction

Ovarian cancer (OvCa) is a disease with limited screening strategies for early diagnosis, usually presenting in the advanced stage when first diagnosed, with a high mortality rate, even after treatment [1,2]. Epithelial ovarian cancer (EOC) is the most common form of OvCa with 21,750 new diagnoses and 13,940 related deaths in the United States in 2020 [3]. In Taiwan, 1521 new patients were diagnosed in 2017, with an incidence rate of 9.2 per 100,000 person-years, and was ranked the seventh most common form of female cancer in 2017 [4]. High-grade serous carcinoma (HGSC) is the most prevalent and fatal type, accounting for 60% of EOC diagnoses and 60% of female cancer mortalities [3]. Due to the diagnostic dilemma of EOC, several studies on the effective prevention of EOC have been conducted, highlighting opportunistic salpingectomy (OS) as one of them [5,6,7].

Currently, serous tubal intraepithelial carcinoma (STIC) at the fimbria end of the fallopian tubes is thought to be the precursor of HGSC [3,8,9]. Based on this finding, an OS could prevent HGSC. It has been reported that undergoing an OS is associated with reduced OvCa risk [10,11,12]. In our hospital, since 2007, we have implemented OS in our eligible patients [13]. In 2019, the American College of Obstetrics and Gynecology committee suggested performing an OS at the time of a hysterectomy or pelvic surgery as a benign indication for the primary prevention of OvCa [14]. In addition to the prevention of OvCa, undergoing an OS is not associated with increased perioperative complications, such as increased hospitalization days, blood transfusion requirements, nor postoperative infections, and it is cost-effective [15]. Previous studies have revealed no difference in the long-term ovarian function of concurrent OS during benign gynecologic surgeries, measured through the change in anti-Müllerian hormone (AMH), follicle-stimulating hormone (FSH), and antral follicle count (AFC) postoperatively for three months to five years [16,17]. Owing to the possibility that OS might prevent OvCa, the prevalence of concurrent OS with a hysterectomy has increased ten-fold in the last decade [7].

The ovarian function may be hampered by the cauterization of the mesosalpinx during an OS with damaged blood flow to the ovary. Two techniques of OS are reported, including suture ligation and bipolar electrocautery [18]. However, which technique damages the blood flow to the ovary is unknown. The most-used method in laparoscopic hysterectomy is bipolar electrocautery [13,19].

However, the longest follow-up period after an OS is only 5 years [16]. Consequently, a longer follow-up period to assess ovarian function is required. Notably, the impact of an OS on ovarian function may be to cause early menopause and increase the risk of cardiovascular disease (CVD), metabolic syndrome, osteoporosis, and all-cause mortality [20,21]. The objective of this study was to follow patients who underwent a hysterectomy with or without an OS for 5 to 10 years to analyze the effect of an OS on the onset time of menopause.

## 2. Materials and Methods

### 2.1. Data Sources

We used the hospital’s electronic medical history database to retrieve data on patients who had undergone a hysterectomy with or without an OS over a 9-year period (January 2007 to December 2015) and who were followed-up until July 2021.

### 2.2. Study Design and Participants

This was a retrospective cohort study. We used the health insurance surgical code payment system (hysterectomy: 80416B, 80421B, 80404B, 80403B) in Taiwan to recruit patients who had undergone a hysterectomy in our hospital from 1 January 2007 to 31 December 2015.

The inclusion criteria were females aged 20 to 50 years who had undergone a hysterectomy for benign lesions, including endometriosis, leiomyoma, and prolapse, and who were followed-up until 31 July 2021.

The exclusion criteria were as follows: patients aged >50 years; those with malignant pathology, including uterine, ovarian, or cervical malignancy; partial or complete adnexectomy; loss of baseline characteristic data; and those with menopause before the operation. Menopause before the hysterectomy was defined as amenorrhea for one year according to the admission and nursing note records.

Patients were divided into three subgroups based on age: <40, 40–44, and 45–50 years old. Baseline demographics were collected, including the indication for hysterectomy, surgical approach (open, laparoscopic, or vaginal), and underlying disease such as diabetes mellitus (DM), hypertension, body mass index (BMI), and parity.

### 2.3. Outcome Measures

Menopause without hysterectomy is defined as amenorrhea for 12 months [22]. However, the diagnostic criteria for menopause in females undergoing a hysterectomy cannot be evaluated based on amenorrhea. Therefore, supportive criteria that can be used to assess menopause in post-hysterectomy patients, including menopausal symptoms, biochemical data, and hormone replacement therapy, were used [23]. In our study, patients with one of the following menopausal criteria were regarded as having menopause: diagnosis of menopause (International Classification of Diseases (ICD) 9/10 627.2/N95.1), those with menopausal symptoms, receiving menopausal hormone therapy (HT), and those with FSH levels of >40 IU/L or low levels of AMH [24,25].

### 2.4. Statistical Analysis

Student’s *t*-tests were used to compare continuous variables. Fisher’s exact tests were used to compare the categorical variables. Statistical analyses were conducted using SPSS 25.0 (IBM, Armonk, NY, USA). The linear regression was used to analyze the association between times from surgery to menopause and covariates, presented as β and 95% CIs. We used G*Power 3.1.9.2 to calculate the sample size. To compare the mean difference in time from surgery to menopause between the OS and non-OS groups, we set an effect size of 0.7, α of 0.05, power(1-β) of 0.80, OS to non-OS sample size ratio of 2, and a two-sided test was performed. The estimated sample size was 76 (i.e., 51 and 25 in the OS and non-OS groups, respectively). Statistical significance was set at a *p*-value of <0.05.

## 3. Results

### 3.1. Demography

**Figure 1:**
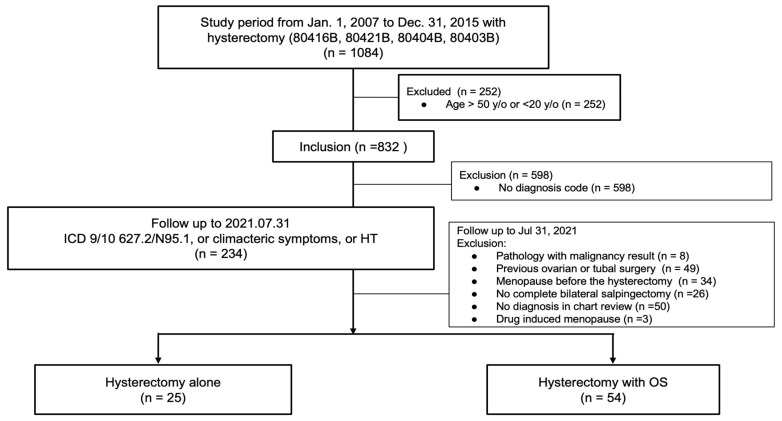
Flow chart of study design. OS: opportunistic salpingectomy; HT: hormone therapy.

The search strategy described above retrieved 1084 patients. Overall, 79 patients were included in the study: 45 in the OS group and 25 in the non-OS group (Figure 1). Table 1 shows the demographic characteristics of the patients. The mean age at the time of surgery, parity, hypertension, DM, surgical indications, and HT were not significantly different between the groups. However, BMI was significantly different (OS: 25.27 ± 4.17 vs. non-OS: 22.97 ± 3.27, *p* = 0.01).

### 3.2. Menopause Characteristics of the Patients

Among menopausal symptoms, only sleep problems were more prevalent in the OS group than in the non-OS group (41% vs. 12%, respectively, *p* = 0.01) (Table 2).

### 3.3. Onset Time of Menopause after Surgery

Table 3 shows the menopausal onset of the patients in both groups. There was no difference in the age at surgery (45 years old), nor at menopause between the two groups. However, the duration from surgery to menopause was significantly shorter in the OS group than in the non-OS group (*p* = 0.031).

### 3.4. Subgroup Analysis

A subgroup analysis between menopause and OS in different age groups was performed (Table 4). There were no significant differences in the onset time from surgery to menopause between the OS and non-OS groups based on age.

### 3.5. Analysis after Adjusting Covariates

A linear regression analysis of time from surgery to menopause considering various covariates was performed (Table 5). The age at surgery, BMI, and parity had no association with time from surgery to menopause. The OS was associated with a significantly shorter period between surgery and menopause (*p* = 0.029).

## 4. Discussion

Our study reviewed 79 patients, 54 in the hysterectomy accompanied with OS group and 25 in the hysterectomy-only group. We found that a high BMI and menopausal sleep problem symptoms were more prevalent in the OS group. Additionally, the duration from surgery to menopause was significantly shorter in the OS group than in the non-OS group.

### 4.1. The Association between Hysterectomy, OS, and Ovarian Function

A hysterectomy can decrease ovarian function by disrupting ovarian blood flow, causing follicular depletion [25,26]. Recently, a concurrent OS with hysterectomy has been shown to significantly decrease the risk of OvCa due to the elimination of the fallopian tubes, the origin of HGSC which contains STIC, TP53, and BRCA1/2 mutations [3,27]. Most studies have supported that an OS does not increase perioperative complications nor decrease long-term ovarian function (with a <5-year follow-up) [10,14,17]. Conversely, our study might reveal a negative association between an OS and early menopause, compared to that of the non-OS group. This difference between ours and previous studies may be due to the longer follow-up times. We enrolled patients over a 9-year period and followed-up on them for more than 5 years. The mechanisms underlying the negative associations between an OS and long-term ovarian function remain unclear and require further long-term follow-up studies [28].

The evaluation of menopause is difficult because of the lack of menstruation after a hysterectomy [23]. Most studies that have evaluated the effect of an OS on ovarian function were based on blood tests measuring AMH, AFC, estradiol, and FSH [29]. However, these biochemical methods for diagnosing menopause have limited value due to the long-term continuous fluctuation of hormones during perimenopause to menopause status, with no definitive biochemical levels for diagnosing menopause [30]. Our study used diagnostic codes, biochemical analysis, HT requirement, and the patients’ self-reported menopausal symptoms to characterize menopause.

### 4.2. Population Study Regarding OS and Ovarian Functions

Collins et al. conducted a register-based cohort study and recruited 4906 patients between 1988 and 2016 who had undergone a hysterectomy for benign diseases with or without an OS [31]. This was the first study to evaluate long-term ovarian function based on the presence of postmenopausal symptoms for one year postoperatively. The results showed that an OS increased the risk of postmenopausal symptoms (adjusted relative risk 1.33, 95% confidence interval 1.04–1.69) [31]. Chan et al. performed a prospective observational study recruiting 32 patients who had received salpingectomy and also revealed that a salpingectomy impacted ovarian function by observing patients who had undergone salpingectomies for ectopic pregnancies. They found that AFC and 3D Doppler ovarian blood flow were significantly decreased at the salpingectomy site when compared to the corresponding normal side [32].

### 4.3. Systemic Review Regarding OS and Ovarian Cancer Prevention

The previous systemic review discussed the effect of preventing ovarian cancer after an OS during a hysterectomy [33]. They included 11 studies and found the hazard ratio was 0.65 (95% CI: 0.52–0.81) and the odds ratio was 0.58 (95% CI: 0.36–0.95) for ovarian cancer risk after an OS. Two randomized trials and one observational study showed no influence on ovarian endocrine function after an OS group compared with a non-OS group [33,34,35,36]. However, they concluded that the evidence level was very low for all outcomes. Conversely, our study found that a reduced ovarian function might cause early menopause after an OS group was compared with a non-OS group. Our results need to be confirmed by a large scale well-designed randomized controlled trial.

### 4.4. The Age Effect on the Association between OS and Ovarian Function

Collins et al. also found females aged 44–49 years had a higher menopausal risk than younger females [31]. The earlier onset of menopause in the OS group may be due to their older age, in which they are closer to a perimenopause or menopause status when compared to younger age groups [31]. More long-term follow-up investigations are required in these younger age groups [31,37]. Our results showed no significant difference among the three age groups at 5- and 10-year follow-ups. Nevertheless, our study revealed a trend of shorter periods from surgery to menopause in the 40–44-year-old group. Further large-scale studies are required to elucidate the underlying mechanisms behind this trend.

### 4.5. BMI Effect on the Association between OS and Ovarian Functions

A previous retrospective observational study that recruited 1822 patients showed a mean BMI of 24.18 ± 3.78 kg/m^2^ (overweight) among Chinese women who underwent a salpingectomy for a benign disease [38]. Collins et al. reported no difference in BMI between an OS (26.5 ± 4.5 kg/m^2^) and non-OS group (26.4 ± 4.7 kg/m^2^) among Swedish females [31]. However, the relative risk of early menopausal symptoms increased after adjusting for BMI (1.03, 95% CI: 1.01–1.06) [31]. In our study, the BMI was significantly higher in the OS group (25.27 ± 4.17) than in the non-OS group (22.97 ± 3.27). The association between BMI and early menopause in patients of different ethnicities who have undergone an OS requires further exploration.

### 4.6. The Trends of OS

Since EOC originates from the fallopian tubes, we have been performing an OS concurrently with hysterectomies for benign indications since 2007, with a prevalence of 8% in 2007, which rapidly increased to 80% in 2015 [13].

### 4.7. Strength and Limitation of the Study

A strength of our study is the long-term 5–10-year follow-up data we have generated, in addition to biochemistry, menopausal symptoms, and HT requirement data. These measurements support a greater clinical association and precise diagnosis of menopause. Additionally, we can continue to follow-up on the younger group to an age which is closer to menopause to improve the limitations of the previous studies. We also collected baseline characteristics to address the confounding factors that can contribute to decreasing ovarian function, such as smoking, BMI, hypertension, and DM [39,40].

A limitation of this study is the small patient cohort. Additionally, the retrospective nature of this study also showed that patient characteristics were as detailed as desired, and the risks of OvCa for each patient were unknown. There is no standard definition of menopause for females undergoing a hysterectomy, meaning different studies have used various biochemical methods and clinical presentations to diagnose menopause. Menopausal symptoms were recalled by the patients, meaning recall bias may have affected this study.

## 5. Conclusions

In conclusion, our study showed that an OS during a hysterectomy might cause earlier menopause than a hysterectomy only. The consequence of possible earlier menopause might have caused an increase in morbidity and mortality rates, such as CVD, metabolic syndrome, and osteoporosis. Surgeons need to share decision-making with the patient, particularly those with a low risk of OvCa, regarding the benefits and possible impacts of an OS before surgery. These findings require further large-scale studies to reinforce this relationship.

## Figures and Tables

**Table 1:** Patient demographics between both groups.

Item		Surgery		Total	*p*-Value
		OS	Non-OS		
N		54	25	79	
Age at the time of surgery		45.52 ± 3.2	45.76 ± 3.47	45.59 ± 3.26	0.769
Age group					0.462
	<40 y/o	4 (7.4%)	1 (4%)	5 (6.3%)	
	40–44 y/o	9 (16.7%)	7 (28%)	16 (20.3%)	
	45–50 y/o	41 (75.9%)	17 (68%)	58 (73.4%)	
BMI (kg/m^2^)		25.27 ± 4.17	22.97 ± 3.27	24.54 ± 4.03	0.01 *
BMI group					0.041 *
	Underweight	1 (1.9%)	1 (4%)	2 (2.5%)	
	Normal	22 (40.7%)	18 (72%)	40 (50.6%)	
	Overweight	14 (25.9%)	4 (16%)	18 (22.8%)	
	Obese	17 (31.5%)	2 (8%)	19 (24.1%)	
Parity		2.26 ± 1.09	2.24 ± 1.23	2.25 ± 1.13	0.947
HTN		3 (5.6%)	1 (4%)	4 (5.1%)	0.623
DM		1 (1.9%)	2 (8%)	3 (3.8%)	0.234
Approach					0.279
	Laparoscopic	31 (57.4%)	12 (48%)	43 (54.4%)	
	Abdominal/open	23 (42.6%)	12 (48%)	35 (44.3%)	
	Vaginal	0 (0%)	1 (4%)	1 (1.3%)	
Indication					0.513
	Leiomyoma	28 (51.9%)	16 (64%)	44 (55.7%)	
	Endometriosis	25 (46.3%)	9 (36%)	34 (43%)	
	Prolapse	1 (1.9%)	0 (0%)	1 (1.3%)	
HT use		39 (72.2%)	19 (76%)	58 (73.4%)	0.724

Data are presented as n or mean ± standard deviation or n (%). * *p*-value < 0.05 was considered statistically significant after test. OS: opportunistic salpingectomy; HT: hormone therapy; HTN: hypertension; DM: diabetes mellitus; BMI: body mass index; y/o: years old.

**Table 2:** Menopause characteristics of the patients.

Item		Surgery		Total	*p*-Value
		OS	Non-OS		
HT use		39 (72.2%)	19 (76%)	58 (73.4%)	0.724
Menopause symptoms					
	Hot flush	21 (38.9%)	12 (57.1%)	33 (44%)	0.153
	Night sweats	12 (22.6%)	5 (25%)	17 (23.3%)	0.529
	Sleep problems	7 (13%)	9 (41%)	16 (21.1%)	0.01 *
	Mood changes	4 (7.5%)	0 (0%)	4 (5.6%)	0.285
	Dryness	6 (11.3%)	3 (15.8%)	9 (12.5%)	0.440
	Dysuria	3 (5.6%)	0 (0%)	3 (4.1%)	0.399
	Recurrent UTI	0 (0%)	0 (0%)	0 (0%)	1
	Sexual dysfunction	7 (13%)	1 (5.3%)	8 (11%)	0.328

Data are presented as n or mean ± standard deviation or n (%). * *p*-value < 0.05 was considered statistically significant after test. UTI: urinary tract infection; OS: opportunistic salpingectomy.

**Table 3:** Postoperative onset time of menopause.

Item	Surgery		Total	*p*-Value
	OS (*n* = 54)	Non-OS (*n* = 25)		
N	54	25	79	
Age at surgery	45.52 ± 3.2	45.76 ± 3.47	45.59 ± 3.26	0.769
Age at menopause	47.36 ± 3.9	48.69 ± 3.96	47.78 ± 3.94	0.165
Surgery to menopause time (years)	1.84 ± 1.85	2.93 ± 2.43	2.18 ± 2.09	0.031 *

Data are presented as mean ± standard deviation. * *p*-value < 0.05 was considered statistically significant after the test. OS: opportunistic salpingectomy.

**Table 4:** Comparison of the length of time from surgery to menopause between the different surgery groups stratified by age.

Age Group	Item	Surgery		Total	*p*-Value
		OS (*n* = 54)	Non-OS (*n* = 25)		
<40 y/o (*n* = 5)	Surgery to menopause time (years)	0.44 ± 0.40	4.60 ± NA	1.27 ± 1.89	NA
		(*n* = 4)	(*n* = 1)		
40–44 y/o (*n* = 16)		0.80 ± 0.75	2.86 ± 2.71	1.60 ± 2.09	0.094
		(*n* = 9)	(*n* = 7)		
45–50 y/o (*n* = 58)		2.21 ± 1.95	2.86 ± 2.42	2.40 ± 2.10	0.286
		(*n* = 41)	(*n* = 17)		

Data are presented as mean ± standard deviation. OS: opportunistic salpingectomy.

**Table 5:** Factors associated with surgery to menopause time.

Item	Crude		Adjusted	
	β (95% CI)	*p*-Value	β (95% CI)	*p*-Value
Age at surgery	0.02 (−0.12, 0.17)	0.747	0.02 (−0.13, 0.16)	0.814
BMI	0.00 (−0.12, 0.12)	0.998	0.04 (−0.09, 0.16)	0.563
Parity	0.04 (−0.38, 0.46)	0.844	0.04 (−0.38, 0.46)	0.856
Surgery (OS vs. non-OS)	−1.09 (−2.07, −0.1)	0.031 *	−1.16 (−2.21, −0.12)	0.029 *

CI: confidence interval; BMI: body mass index; OS: opportunistic salpingectomy. * *p*-value < 0.05 was considered statistically significant after test.

## Data Availability

All data are presented in the manuscript.
